# Optimizing thermal diffusivity measurements for fluids with accessible 3D printing and Arduino-based temperature control

**DOI:** 10.1016/j.ohx.2025.e00646

**Published:** 2025-04-04

**Authors:** Miguel Ceja-Morales, Pedro E. García-González, Luis M. Montes-De-Oca, R.A. Medina-Esquivel, Miguel Zambrano-Arjona, Nikte M. Gomez-Ortiz, P. Martínez-Torres

**Affiliations:** a Instituto de Física y Matemáticas, Universidad de Michoacan of San Nicolas de Hidalgo, Edificio C3-C, Ciudad Universitaria, Michoacan, Mexico; b Universidad Autónoma de Yucatán, Facultad de Ingeniería, Av. Industrias No Contaminantes, Periférico Norte, Cordemex, Mérida, Yucatán, 150, Mexico; c Universidad Intercultural Indígena de Michoacán, km 3 carretera Pátzcuaro-Erongarícuaro, C.P. 58010, Pátzcuaro, Michoacán, Mexico

**Keywords:** Fluid thermal diffusivity, Additive manufacturing, Arduino temperature control, Cost-effective scientific tools, Thermal wave resonator cavity

## Abstract

This study introduces an instrument to measure thermal diffusivity in fluids, called a Thermal Wave Resonator Cavity, constructed via additive manufacturing (3D printing) and significantly improved by integrating a temperature control system developed with an Arduino microcontroller. The device was assessed through measurement of the thermal diffusivity of distilled water both with and without temperature control. The results demonstrate that the temperature-controlled system yields significantly more reliable and reproducible thermal diffusivity measurements compared to the uncontrolled system. Furthermore, measurements of water’s thermal diffusivity at various temperatures corroborated values previously reported in the literature. This cost-effective and innovative solution leverages accessible technology to enhance the accuracy of thermal measurements, thereby democratizing access to traditionally expensive, high-quality scientific instruments. This approach has the potential to broaden research capabilities across various scientific disciplines by melding affordability with precision.

## Graphical abstract

**Figure:**
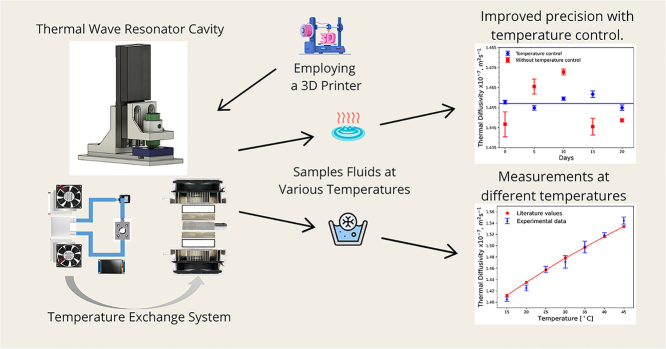

## Specifications table

**Table:** 

Hardware name	*Thermal Wave Resonator Cavity assembled with 3D printer material and temperature controller by Arduino.*
Subject area	*Engineering and material science.*
Hardware type	*Measuring physical properties and in-lab sensors*
Closest commercial analog	*“No commercial analog is available”.*
Open source license	*Mendeley data.*
Cost of hardware	$300homemade parts.
Source file repository	http://dx.doi.org/10.17632/wzvw4zbhzh.2

## 1 Hardware in context

The study of thermal properties in fluids is essential in modern science and technology [1], [2], [3], [4]. This importance stems from their influence on a wide range of applications, from enhancing energy efficiency to developing novel materials. However, accurate characterization of these properties often requires a delicate balance between cost and quality. Existing techniques frequently necessitate a compromise, forcing researchers to choose between expensive, high-precision instruments and more affordable options that may yield less reliable results. To address the critical need for thermal characterization in fluids, a variety of techniques have historically been developed, including calorimetric methods, thermal analysis, and conductivity measurements. Among these, photothermal (PT) techniques are distinguished by their versatility, precision, and effectiveness in characterizing a wide range of materials. These techniques, which exploit the well-known photothermal effect, measure the optical absorption and thermal properties of materials by converting absorbed optical energy into heat [5], [6], [7].

Photothermal techniques have shown their successful applicability for the non-destructive thermal characterization of a wide range of materials. They measure the material’s optical absorption and thermal properties by converting the absorbed optical energy (and not lost by emission) into heat. PT techniques operate under non-steady state conditions with small temperature variations in the sample induced by an external source that can be pulsed, continuous, or modulated. These techniques allow quantifying the thermal field as a function of the sample thickness, time, or frequency.

In some cases, photothermal techniques could be complex and require overwhelming mathematical models. However, the Thermal Wave Resonator Cavity (TWRC) is a precise and practical PT technique for determining thermal diffusivity in liquids. TWRC’s simplicity comes from its ability to isolate thermal diffusion, ignoring radiative and convective effects. The TWRC technique is based on thermal wave theory [8]. It postulates that heat waves generated by an external source propagate through the material and can be analyzed to determine its thermal properties [9], [10], [11]. In practice, TWRC works by heating one face of the sample with a modulated laser beam, allowing thermal waves to propagate through the material until they reach a temperature sensor on the opposite face. The thermal signal captured by the sensor is analyzed and compared with analytical solutions to determine the thermal diffusivity of the material. Unlike other PT techniques, TWRC is simpler to handle because the modulation frequency of the excitation source remains constant. Since the sample is a fluid, a scan can be carried out through its thickness, obtaining an exponential decay in the signal amplitude and a linear phase shift between the heat source and the temperature sensor. On a logarithmic scale of the signal amplitude, this data is represented as a straight line as a function of the thickness of the sample. On a linear scale of the phase shift, a linear behavior is also obtained as a function of the sample thickness. This significantly simplifies the mathematical model since a transfer function as a frequency function is unnecessary. TWRC is a very user-friendly technique that offers high precision and accuracy.

The TWRC technique has been helpful in the thermal characterization of different fluids, including mixtures of two solvents and nanofluids composed of metallic, ceramic, magnetic, and semiconductor nanoparticles [12], [13], [14], [15]. Furthermore, the technique has demonstrated great versatility, allowing studies of magnetorheological fluids and magnetic nanofluids when placed within a Helmholtz coil system, thanks to its compact size [16]. It has also been used to obtain the thermal properties of thin films [17]. However, the thermal properties of some materials strongly depend on their surrounding temperature, especially those that undergo phase transitions near the temperature of the environment where the experiment is conducted, such as surfactant solutions, hydrogels, and emulsions [18]. This temperature sensitivity can lead to significant variations in the measured thermal properties, reducing the accuracy and precision of the measurements. Additionally, there is a gap in the accessibility of high-quality prototypes for thermal property measurements, particularly for labs with limited resources. The democratization of access to these tools is essential. The integration of 3D printing technology allows for the creation of customized, cost-effective prototypes, while the implementation of homemade, accessible, and high-quality instrumentation for conditioning the signal from the pyroelectric sensor addresses a crucial need in this field.

This article details the development of a temperature control system integrated with the TWRC device, which was fabricated using 3D printing. The use of 3D printing technology allowed for rapid and cost-effective production, enabling customization of the device’s components to fit specific experimental needs. This flexibility not only reduces production costs but also minimizes waste, making it an efficient and environmentally friendly approach. The system, which includes Peltier cells, an Arduino Mega microcontroller with PID control, and a water pump, effectively stabilizes the sample temperature. This stabilization is crucial for improving measurement precision and enabling detailed thermal diffusivity studies across various temperatures.

Additionally, signal conditioning stages were implemented before the lock-in detection process to enhance the signal-to-noise ratio. These stages are vital for filtering and amplifying the signal, ensuring the data collected is accurate and reliable. The integration of 3D printing and advanced signal conditioning into the TWRC device forms a cohesive, highly functional system. This method allows for rapid, cost-effective production with customizable components, making the device ideal for labs with limited budgets. This advancement democratizes access to precise thermal characterization tools, offering comparable accuracy to more expensive equipment and potentially accelerating research in various scientific fields.

## 2 Hardware description

**Fig. 1:**
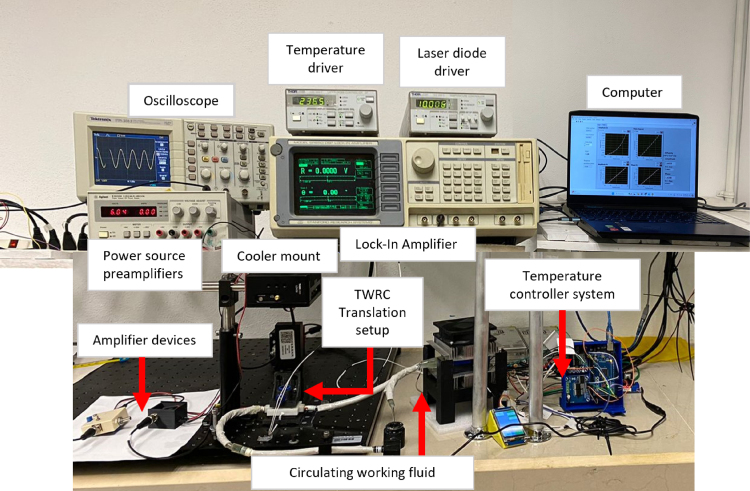
Schematic diagram of the thermal wave resonant cavity setup.

The TWRC has become a well-established technique for determining the thermal diffusivity of fluids [19]. Although the implementation of this technique often involves specialized equipment such as a lock-in amplifier, signal preamplifier, motorized translation stage, and laser driver, in this project, it was possible to build a high-quality experimental apparatus using 3D-printed components and conventional electronic components such as a preamplifier and a charge amplifier. This implementation can reduce costs and facilitate its development in laboratories to perform thermal diffusivity measurements. Additionally, a temperature controller system was built to set the temperature of the study samples to a desired value in the range from 15 °C to 45 °C. The temperature control device was developed using a closed recirculation system connected to the TWRC reservoir. The cost of this system was less than US $300, which included the pump, hoses, refrigeration system, Arduino board, and the other electronic consumables and devices used. Fig. 1 shows the complete view of the implemented TWRC. The homemade parts are indicated with arrows, which will be described further.

### 2.1 Thermal wave resonator cavity technique

**Fig. 2:**
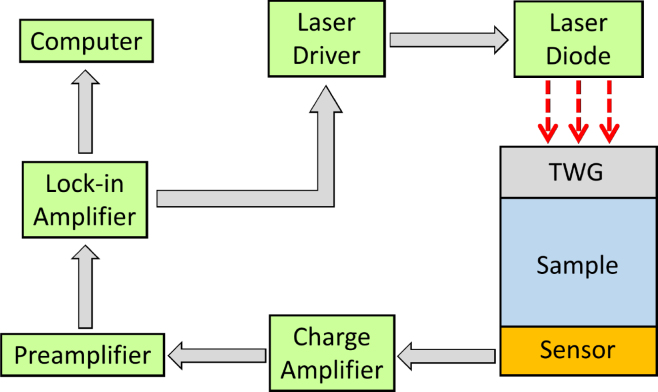
Schematic diagram of the thermal wave resonant cavity setup. TWG refers to thermal wave generator.

Fig. 2 shows the block diagram of the TWRC experimental setup. It consists of a laser driver to control a laser diode current and a lock-in amplifier to generate a reference sinusoidal signal that is sent to the laser driver to modulate the laser light intensity output sinusoidally (dashed arrows in Fig. 2). This electromagnetic radiation reaches the thermal wave generator (TWG), a silicon wafer acting as an optical-to-thermal power converter. The thermal wave generator is in contact with the sample, and the heat is propagated through the sample by conduction to the pyroelectric sensor placed on the opposite side. The pyroelectric signal is sent through a charge amplifier and a preamplifier before being sent to the lock-in amplifier. Finally, the lock-in amplifier decomposes this signal into amplitude and phase and records it on a computer.

### 2.2 Theory of photothermal signal measurement

To develop an instrument capable of determining the thermal property of a liquid sample, it is essential to find the dependence of the pyroelectric signal generated by the thermal wave on the thermal diffusivity of the fluid sample and the cavity length.

**Fig. 3:**
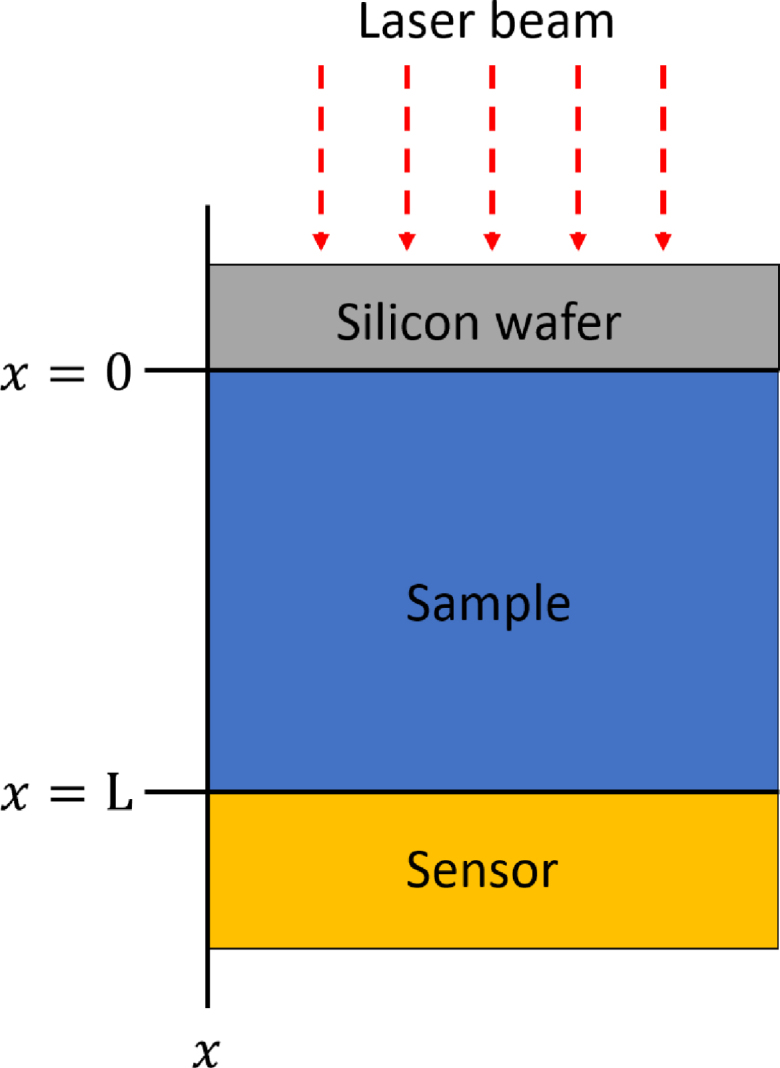
Detailed configuration of the sample chamber in the thermal wave resonant cavity system.

Fig. 3 shows the geometrical configuration of the thermal wave resonator cavity. The temperature field in the regime of the experimental measurements is determined through a practical approximation, considering one-dimensional heat diffusion in one single layer (sample layer in Fig. 3), with its justification discussed in this section. Thus, after the optical-to-thermal conversion of the modulated laser light by the silicon wafer, the thermal wave is assumed to be propagated in one dimension through the fluid from x=0 to x=L, where the sensor is located, and the temperature field distribution follows the heat diffusion equation:

(1) $$\frac{\partial^{2}T\left(x,t\right)}{\partial x^{2}}-\frac{1}{\alpha }\frac{\partial T\left(x,t\right)}{\partial t}=0,$$

where T is the temperature field function, and α is the thermal diffusivity of the sample. The Eq. (1) is subjected to the boundary condition given by the heat flux produced by the modulated laser beam as $-k\frac{dT\left(x=0\right)}{dx}=F_{0}\left[\cos\left(\omega t\right)\right]=F_{0}\text{Re}\left[\exp\left(i\omega t\right)\right]$ and the adiabatic boundary condition $\frac{dT\left(x=L\right)}{dx}=0$, where $i=\sqrt{-1}$, k the thermal conductivity of the sample and ω=2πf with f the modulated laser frequency. Thus, the solution of Eq. (1) at x=L is [10]:

(2) $$T\left(x=L,t\right)=\frac{F_{0}}{2k\sigma }\left(\frac{e^{-\sigma L}}{1-e^{-2\sigma L}}\right)e^{i\omega t}$$

where $\sigma =\left(1+i\right)\frac{1}{\mu }$ with $\mu =\sqrt{\frac{\alpha }{\pi f}}$ the thermal diffusion length.

Eq. (2), beyond being a simplified expression, accurately represents the temperature field when the “thermally thick” condition is satisfied, where the sample thickness exceeds twice the thermal diffusion length (L>2μ). Under this approximation, the complex voltage signal of the sensor is:

(3) $$V\left(L,\alpha ,f\right)=F\left(f\right)T\left(x=L,t\right)=F\left(f\right)e^{-\left(i+1\right)\frac{L}{\mu }}e^{i\omega t},$$

F(f) is the instrumental transfer function and is constant when the modulated frequency is a constant value. It is important to emphasize that the adiabatic boundary condition used to obtain Eq. (2) does not imply that no heat reaches the sensor experimentally. In the thermally thick regime measurement, the thermal wave is sufficiently attenuated before reaching the sample’s rear surface, rendering the heat flux there negligible relative to the front boundary [9]. Nonetheless, this residual temperature variation remains large enough to produce the pyroelectric response. This reasoning is consistent with the typical photothermal or thermal-wave analyses using the thermally thick approximation [12], [14], [18], where significant attenuation in thick samples validates the adiabatic boundary assumption while preserving the essential physics of the signal generation. For a more detailed description of the thermal field considering several layers, see [10], [19].

This voltage signal can be expressed as V=Aexp(iϕ), where A and ϕ are the voltage amplitude and phase as a function of sample thickness. Thus, the amplitude and phase signal can be separated as $\ln\left|A\right|=C_{v}-\frac{1}{\mu }L$, and $\phi =C_{\phi }-\frac{1}{\mu }L$, that correspond to straight lines, and their slopes are related to thermal diffusivity as:

(4) $$\alpha_{j}=\frac{\pi f}{m_{j}^{2}}$$

where $m_{j}$ is the slope of the experimental data with j={A,ϕ} referring to the amplitude or phase signal, respectively. Hence, the thermal diffusivity is determined by the slopes of the amplitude and phase signals obtained from the pyroelectric sensor for a thickness scan at a fixed frequency.

## 3 Design files summary

**Table:** 

Design filename	File type	Open source license	Location of the file
**TWRC system:**	Fig. 5, PNG	Open-source license	TWRC system
Base plate	CAD file, 3D print	Mendeley license	Base
Angle bracket	CAD file, 3D print	Mendeley license	Angle bracket
Generator cylinder	CAD file, 3D print	Mendeley license	Cylinder
Bracket support	CAD file, 3D print	Mendeley license	Bracket support
Sample reservoir	CAD file, 3D print	Mendeley license	Reservoir
Software TWRC	LabVIEW code	Mendeley license.	LabVIEW code

**Charge amplifier**	Fig. 6, PNG	Open-source license	Charge amplifier

**Amplifier AD620**	Fig. 7, PNG	Open-source license	Amplifier AD620

**Temperature control system:**	Fig. 8, PNG	Open-source license	Temperature control system
Electronic system	Arduino IDE	Open-source license	Arduino code
Nextion system	Arduino IDE	Open-source license	Arduino Nextion code

**TWRC system** refers to the parts fabricated on a 3D printer and the CAD files localization, and **Software TWRC** refers to the code developed in LabVIEW software. **Amplifier AD620** and **Charge amplifier** are the commercial and homemade amplifiers used in the experimental setup. **Temperature control system** presents the parts to be assembled and the Arduino codes.

## 4 Bill of materials summary

**Table:** 

Designator	Component	Number	Cost per unit (US$)	Total cost	Source of materials	Material
A2	Angle bracket	1	$5.0	$5.0	3D-printed	PETg
A3	Bracket support	1	$5.0	$5.0	3D-printed	PETg
A4	Generator cylinder	1	$5.0	$5.0	3D-printed	PETg
A5	Silicon wafer	1	$20.0	$20.0	ebay.com	Silicon
A6	Sample reservoir	1	$5.0	$5.0	3D-printed	PETg
A7	Piezoelectric sensor	1	$5.0	$5.0	amazon.com	Electronic
A8	O-ring	1	$2.0	$2.0	amazon.com	Nitrile

B1	Amplifier module AD620	1	$9.0	$9.0	amazon.com	Electronic

C1	Operational amplifier TL081	1	$1.5	$1.5	amazon.com	Electronic

D1	Arduino Mega	1	$20.0	$20.0	amazon.com	Electronic
D2	Source 12 V	2	$23.0	$46.0	amazon.com	Electronic
D3	H-Bridge	2	$17.5	$35.0	amazon.com	Electronic
D4	Peltier cell	2	$6.0	$12.0	amazon.com	Electronic
D5	Aluminum plate	2	$6.0	$12.0	amazon.com	Aluminum
D6	Fan 12 V	2	$8.0	$16.0	amazon.com	Electronic
D7	Heat exchanger chamber	1	$8.0	$8.0	amazon.com	Aluminum
D8	Water pump	1	$8.0	$8.0	amazon.com	Electronic
D9	Nextion touch screen	1	$25.0	$25.0	amazon.com	Electronic
D10	Thermistor NTC 100k Ohm	4	$2.5	$10.0	amazon.com	Electronic

## 5 Build instructions

### 5.1 Overall TWRC setup

**Fig. 4:**
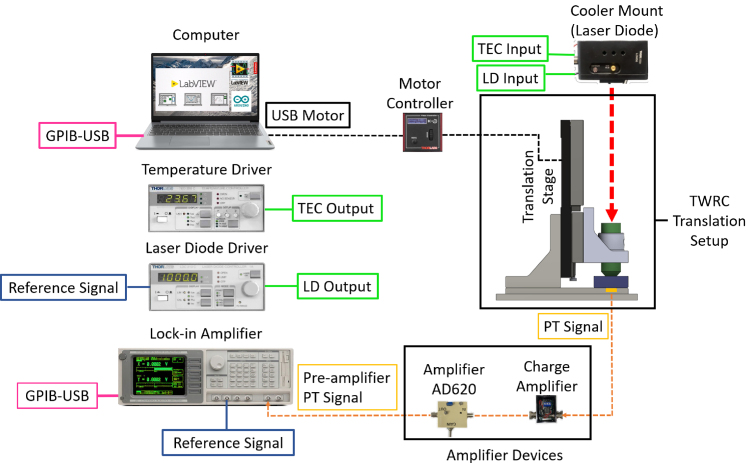
Schematic diagram of the thermal waver resonator cavity setup.

Fig. 4 shows a complete view of the experimental setup. The assembly of the entire measurement system begins by connecting the reference signal SINE OUTPUT of the lock-in amplifier (Stanford Research, SR-850) to the MOD IN connector of the laser diode current (LDC) controller (Thorlabs, LDC220C). At the same time, the LDC driver and the laser diode temperature (TED) controller (Thorlabs, TED200C) are connected to a thermoelectric-cooled mount device (Thorlabs LDM90) to regulate the temperature and operating current of an 808 nm laser diode (Thorlabs, L808P1WJ), through the LD/TEC OUTPUT and INPUT connectors, respectively.

Once the laser diode (LD) is chosen to be used in the TWRC experiment, it is important to identify its current threshold and maximum operating current to adjust the modulated power output in the range of LD operation, before starting to assemble the TWRC Translation Setup. This procedure is as follows: a sinusoidal voltage signal with a fixed frequency is generated from the SINE OUT BNC of the lock-in amplifier, whose voltage amplitude is defined by the root mean square of the wave ($V_{rms}$). The LDC controller reads this reference voltage through the MODE IN BNC, which controls the current over the laser. The voltage amplitude fixed in the lock-in amplifier must be calculated so that the LDC controller generates a current waveform whose amplitude must be higher than the threshold current $I_{th}$ and less than the maximum operation current, $I_{f}\leq I_{max}$, of the LD used. The $V_{rms}$ set in the lock-in amplifier is calculated as:

(5) $$V_{rms}=\frac{V_{p}}{\sqrt{2}}=\frac{1}{\sqrt{2}}\frac{I_{f}-I_{op}}{K},$$

where $V_{p}$ is the peak voltage of the sine wave, $I_{op}$ is the central value of the desired current between $I_{th}$ and $I_{f}$, and K is the modulation coefficient of the LDC controller. For the LDC220C Thorlabs controller, K is 200mA/V.

### 5.2 TWRC translation setup

**Fig. 5:**
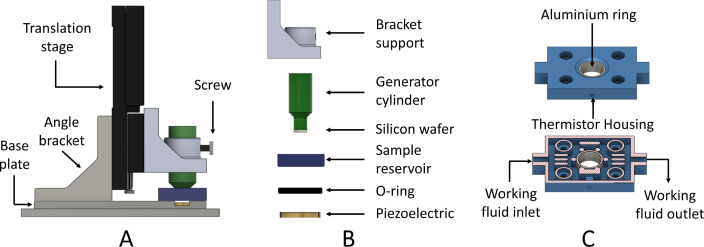
CAD draw of the 1D translator assembled from parts fabricated on a 3D printer.

The part of the TWRC related to the sample’s thickness change and the thermal wave propagation to reach the sensor was developed using a 3D printer with a poly(ethylene terephthalate) glycol (PETg) polymer filament. The five printed parts are shown in Fig. 5. They are labeled as follows: **base plate** that supports the complete structure of the TWRC; **angle bracket** for vertical mounting of the translation stage (Thorlabs, MTS25/M-Z8); **thermal wave generator cylinder** which goes inside the sample to generate the thermal wave through by optical-to-thermal conversion with a silicon wafer at the bottom of the cylinder; **bracket support** of the thermal wave generator, which connects the motorized translation stage to the thermal wave generator cylinder; and the **sample reservoir**, which contains the sample and the working fluid that passes through it to keep the sample at a constant temperature using an implemented homemade temperature control system. Additionally, an O-ring is employed to seal between the sensor and the sample reservoir to prevent fluid leakage.

Fig. 5A shows a view of the arrangement in which the pieces are assembled in the experimental setup, and Fig. 5B shows the order in which the pieces are placed in the TWRC. Careful alignment of the laser diode in the translation direction of the thermal wave generator cylinder is essential, ensuring that the collimated beam aligns precisely with the center of the thermal generator. Additionally, a light-diffusive material layer is attached near the silicon wafer to achieve a top-hat beam intensity radiation from the laser on the silicon wafer to guarantee 1-D heat conduction, as was considered in the solution of Eq. (1).

Fig. 5C shows the internal structure of the sample reservoir, which consists of a cavity through which the working fluid circulates and exchanges heat with an aluminum ring that contains the sample. The columns have the only function to support the container cover.

As previously mentioned, the TWRC measures different sample thicknesses to observe thermal transport through the material. Thus, the thermal wave generator cylinder, mounted on the motorized translation stage, moves vertically to adjust the sample thickness. To precisely control the velocity and acceleration of the thermal wave generator step size inside the reservoir. The motorized translator is interfaced with the computer via a USB connection with its driver Thorlabs KDC101. The sensor is placed at the bottom of the reservoir to measure the resulting thermal waves as a function of the sample’s thickness.

Once the thermal wave reaches the sensor, a PT signal is generated and returned to the lock-in amplifier. In this setup, a piezoelectric sensor detects the PT signal, which acts as a transducer and transforms its thermal expansion into voltage driven by the modulated temperature changes in the sample. Before sending the signal to the lock-in, it is conditioned and preamplified. In this stage, the piezoelectric signal is initially received by a charge amplifier and then processed by an amplifier AD620 module to be returned to the lock-in amplifier (Signal Input - A). All these connections are made with BNC connectors to ensure optimal signal integrity throughout the various stages of the setup.

A homemade Labview program automated a thickness scan, and it acquired the amplitude and phase of the PT signal processed by the lock-in amplifier through a GPIB to USB connection between the lock-in and the computer. Below is a description of the charge amplifier, amplifier AD620 module, and acquisition software. It is worth mentioning that it is a particular configuration, and equivalent devices can replace the lock-in amplifier, laser diode, and laser diode current and temperature controllers.

### 5.3 Charge amplifier

**Fig. 6:**
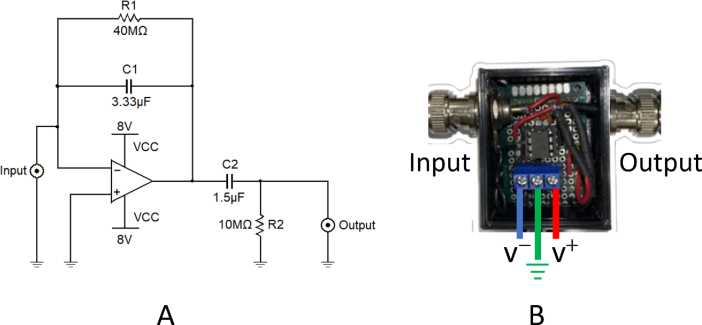
(A) Circuit schematic diagram, and (B) assembled device of the charge amplifier.

A trans-impedance differential charge amplifier is implemented and adapted following [20]. Fig. 6A shows the corresponding schematic diagram, using an operational amplifier, TL081, and additionally, a first-order passive high-pass filter with a cut frequency at 0.01Hz is added as a DC blocking module. With this modification, the obtained sinusoidal signal is stationary and centered at 0 V. Fig. 6A shows the circuit schematic diagram and Fig. 6B shows the implemented physical circuit. This device is coupled between the piezoelectric sensor and the amplifier AD620 module through BNC connectors.

### 5.4 Photothermal signal preamplifier

**Fig. 7:**
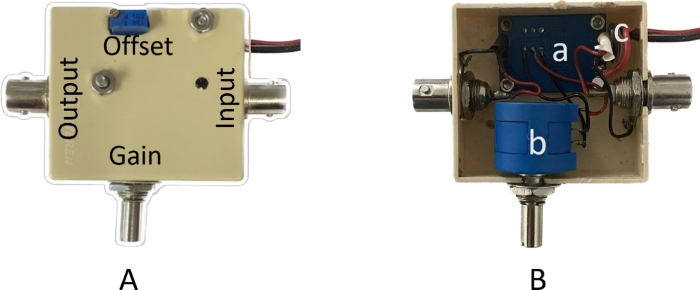
(A) External view of the preamplifier device (B) Internal view with the parts labeled as (a) AD620 module, (b) 100 kΩ potentiometer, (c) 3–12 V DC power input.

A commercial voltage amplifier AD620 module is used to amplify the photothermal signal received from the piezoelectric sensor before being sent to the Lock-in amplifier. This module includes two trimmer potentiometers to adjust the signal’s gain and DC offset. As is shown in Fig. 7B, we replaced the trimmer that adjusts the gain with a multi-turn precision potentiometer to achieve higher control of the piezoelectric amplitude signal and added two BNC connectors for the input and output of the photothermal signal. It operates with a supply voltage ranging from 3 to 12 V, which can amplify the signal generated by the piezoelectric sensor by a factor of up to 1000.

### 5.5 Temperature control system

**Fig. 8:**
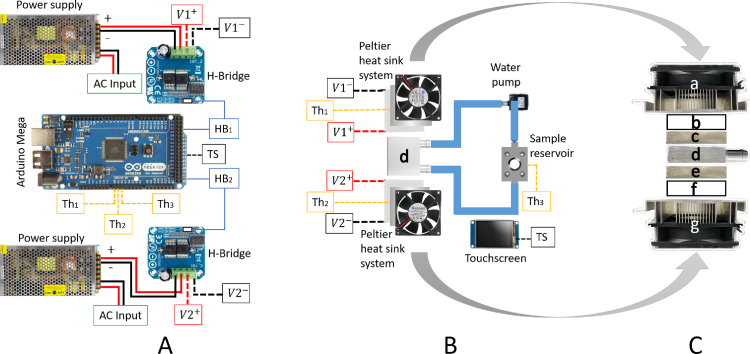
Temperature control system developed with Arduino Mega. (A) Electronic components: power sources, H-Bridge Motor Drivers, Peltier Cells, (B) Peltier heat sink system connected with a water pump to sample reservoir, (C) Peltier heat sink system assembly.

Fig. 8 shows the implemented temperature control system to keep the sample at a constant temperature. It consists of an aluminum heat exchanger chamber in contact with two Peltier cells that exchange heat with a circulating working fluid. The aluminum heat exchanger chamber is connected in a closed-circuit hose to a water pump (AD20P1230E) and to the TWRC sample reservoir, where the working fluid exchanges heat with the sample through an aluminum cylindrical wall (see Fig. 8B). The arrangement of the Peltier cells with the aluminum heat exchanger chamber is shown in Fig. 8C, where the two Peltier cells (b and f) are situated on each side of the heat exchange chamber (d), allowing cooling or heating of the working fluid. Two aluminum plates with an internal cavity (c and e) are placed between them to attach a thermistor on each plate to monitor the temperature of the circulating fluid, and their registered temperature values are used as feedback to perform the PID control. Two fan-cooled heat sinks (a and g) are utilized to dissipate the heat transferred by the Peltier cells.

The temperature control system is implemented in an Arduino Mega microcontroller, where the connections of the electronic components are depicted in Fig. 8A. One BTS7960 H-Bridge (HB) driver for each Peltier cell is connected to the Arduino microcontroller as a power stage of the Peltier cells. These HB drivers are powered with a DC voltage of 12 V. Furthermore, a touch screen (Nextion) was added to facilitate user control of the equipment. Programming codes are included in the open-source Mendeley repository [21].

In Fig. 8A, the thermistors labeled as Th${}_{1}$, Th${}_{2}$, and Th${}_{3}$ are connected to analog pins A0, A1, and A2 of the Arduino. The thermistors Th${}_{1}$ and Th${}_{2}$ are used to perform the PID control of the Peltier cells; the thermistor Th${}_{3}$ monitors the temperature directly in the sample. The Peltier cell voltage supply cables, V$1^{+}$, V$1^{-}$, V$2^{+}$, and V$2^{-}$, are connected to the HB drivers, which in turn are connected to the Arduino (labels HB${}_{1}$ and HB${}_{2}$). For simplicity, Fig. 8A only displays one connection between the eight pins of the HB driver and the Arduino. The eight pins of the HB driver are labeled as VCC, GND, R_IS, L_IS, R_EN, L_EN, RPWM, and LPWM. These pins are connected to the Arduino as follows: VCC and GND are connected to the 5 V and ground pins of the Arduino, respectively. The R_EN and L_EN pins are connected to 5 V, while the RPWM and LPWM pins are connected to two digital pins on the Arduino; in our particular case, we used pin two and pin three for RPWM and LPWM of the HB${}_{1}$ driver, and pin four and pin five for RPWM and LPWM of the HB${}_{2}$ driver. The R_IS and L_IS pins are not connected. The connection of the Nextion touch screen with the Arduino is labeled as TS; again, for simplicity, Fig. 8 only displays one connection. In this case, the touch screen has four pins labeled VCC, GND, RX, and TX and is wired to 5 V, GND, pin 18, and pin 19 of the Arduino Mega, respectively.

**Fig. 9:**
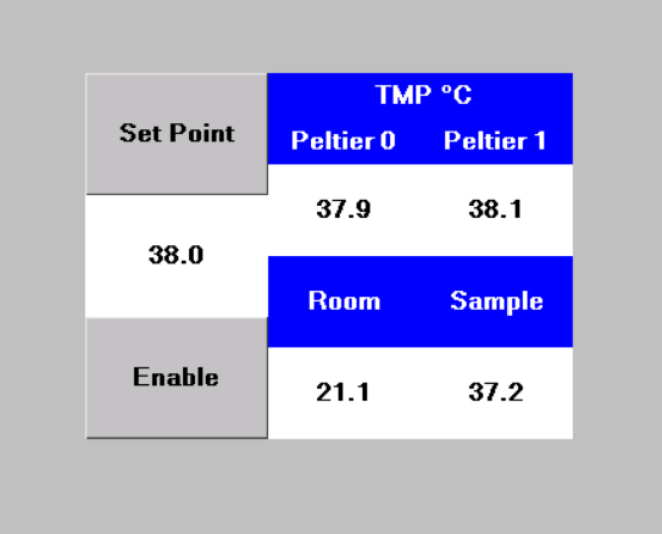
User interface implemented in a Nextion touch screen to set the desired temperature to be controlled by the PID protocol.

Nextion LCD touch screen is a user interface that allows one to fix the temperature set point and monitor the different temperatures of the system. Fig. 9 shows the visual interface that consists of the set point temperature and four temperature values: **Peltier 0** and **Peltier 1** are the temperatures measured at the plates coupled to the Peltier cells (Fig. 8C label as c and e, respectively). The temperature labeled as **Room** refers to the room temperature, and the temperature measured in the sample reservoir is labeled as **Sample**. The protocol for changing the temperature sample consists of selecting a new temperature, tapping on the **Set Point** option, and then entering the value of the new temperature. Subsequently, tap on **Set Point** and then tap on **Enable** option to start the instruction.

### 5.6 PID controller

A PID control is implemented to regulate the sample temperature inside the TWRC sample reservoir. The mathematical representation of the parallel PID control implemented is as follows [22]:

(6) $$u\left(t\right)=K_{p}\cdot e\left(t\right)+K_{i}\cdot \int_{}^{}e\left(t\right)dt+K_{d}\cdot \frac{de\left(t\right)}{dt}$$

where u(t) is the control signal, and $e\left(t\right)=T_{c}-T_{\eta }$ is the control error, which measures the deviation of the actual temperature, $T_{\eta }$, relative to the desired set point, $T_{c}$. The terms in Eq. (6) consist of proportional, integral, and partial derivative operations over e(t). The $K_{p}$, $K_{i}$, and $K_{d}$ parameters are called the proportional, integral, and derivative gains. These parameters were determined using the Ziegler–Nichols open-loop tuning method. This method applies a specific voltage to the Peltier cells, generating a step function of the temperature. The temperature curve over time is recorded, and the PID parameters are determined following the procedure described in [22], [23]. The best parameters obtained using this method were $K_{p}=65$, $K_{i}=6.5$, and $K_{d}=0$.

## 6 Operation instructions

### 6.1 Setting the thickness scan

Before starting an experiment for a particular fluid sample, the user must estimate the value of the thermally thick condition at a fixed frequency used, given by $L=2\mu =2\sqrt{\alpha /\pi f}$, to avoid measuring out of the mathematical approximation. However, it is expected that the thermal diffusivity is unknown for the particular sample. Thus, to determine the range of the thickness scan where the mathematical approximation is valid, an experiment must be conducted in which the value of L gradually increases from zero thickness (thermal wave generator in contact with the sensor) to a large value of L. Then, to identify the range of experimental data that fulfill the thermally thick condition in the Eq. (2), it was done following the methodology proposed by J. Shen et al. [24]. In this methodology, an estimation of the thermal diffusion length (μ) can be computed by direct estimation of the critical points position of the real (In-Phase) and imaginary (Quadrature) parts of the Eq. (2), based on the fact that the thermal waves generated inside the sample are stationary resonant waves with damping, where a good approximation of μ is obtained from the real part of the PT signal by $L_{n}^{IP}=\left(n-\frac{1}{2}\right)\frac{\pi }{2}\mu$, being $L_{n}^{IP}$ the sample thickness positions of the extremes of the PT signal In-Phase (real part) and n represents the index of critical points at that position wave antinode number.

### 6.2 Measurement of thermal diffusivity

**Fig. 10:**
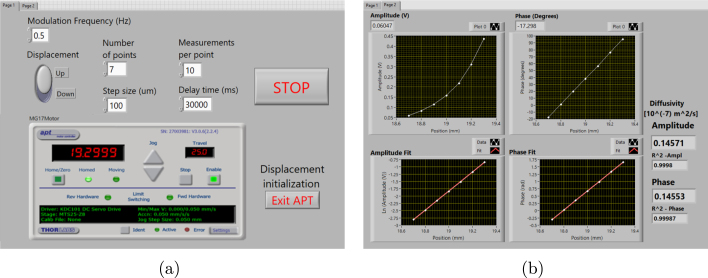
(a) LabView front panel used for operation system. (b) LabView front panel used for data visualization.

To start an experimental measurement, the first step is to place the sample in the TWRC reservoir, fix the bracket support to the platform of the translation stage, and turn on all devices except the LDC. Then, open and run the LabVIEW program, which initializes the control of the stepper motor via the APT Server. Fig. 10(a) provides an overview of the front panel of the automation thickness scan and the Graphical User Interface (GUI) of the APT control of the stepper motor. In the second step, click the “Home/Zero” button on the GUI to set the bracket support to zero (configured by the fault), the largest position relative to the piezoelectric sensor. The next step is to press the digital display above the Home/zero button and set a value on the display to adjust the bracket support until the silicon wafer of the thermal wave generator cylinder contacts the piezoelectric sensor. Once aligned correctly, fasten it securely using the screw on the bracket support (see Fig. 5A). This point will be the origin position of our reference frame, where the sample has zero thickness. At this point, the user already knows the thermally thick condition (L=2μ), which is the position where the measurement starts. In our case, the zero position relative to the sensor corresponds to d=20.000mm from the home position of the translation stage. Thus, the position where the measurement should start is at d−2μ from the home position and finished at d−4μ. The next step is to adjust the travel speed to reduce the vibration of the translation stage and to facilitate the re-accommodation of fluid elements after each movement of the translation stage, which could affect the accuracy of the measurements. To change the travel speed, select the APT Configure Utility by pressing the “Settings” button. In the new window, adjust the velocity and acceleration parameters to $0.05\;{\text{m s}}^{-1}$ and $0.05\;{\text{m s}}^{-2}$, respectively, which works well for us. Save the new parameters and close this window.

The next step is to select the displacement switch in the front panel (above the APT interface) to choose the up or down direction movement of the thermal wave generator. Subsequently, the user writes down the number of points (steps) and the step size of displacement; for instance, in our case, seven points were selected for all measurements, with step sizes of 100μm and a delay time of 30,000ms. The next indicator, “measurements per point”, is the number of measurements per point or position used to calculate the average and error of the PT signal at each measurement point. In the “modulation frequency” box, write down the frequency value selected to perform the laser modulation in the lock-in amplifier; for our particular case, we used 0.5Hz.

Once the translation stage is in position and the TWRC software is configured, turn on the LDC controller and wait at least 10 min for the laser beam and sample temperature to stabilize. After stabilizing, connect the preamplified PT signal to an oscilloscope and adjust the gain of the amplifier AD620 module until achieving an output of around $1\;{\text{V}}_{\text{rms}}$ that is the maximum voltage supported by the lock-in amplifier. This action also helps to show that the signal is sinusoidal and clean during this step. Following signal verification, disconnect the wire from the oscilloscope and connect it to the lock-in amplifier to start with the sample measurement.

The last step is to select the “Exit APT” button to exit the motor controller interface and start the measurement process. This step displays a window where the user can select a file name and store the measured data in a text file. At the same time, a graph is displayed on the second front panel (Fig. 10(b)), which enables monitoring the behavior of the amplitude and phase of the signal during the experiment. The thermal diffusivity value is also determined by the LabView code and displayed in the same frame graphs on one side of the front panel. The “STOP” button halts code execution once the program is running.

## 7 Validation and characterization

The thermal diffusivity of deionized water was measured to validate the TWRC system. The measurements were done with and without temperature control to compare the reliability of the 3D-printed TWRC instrument and the improvement of the measurements with the temperature control.

**Fig. 11:**
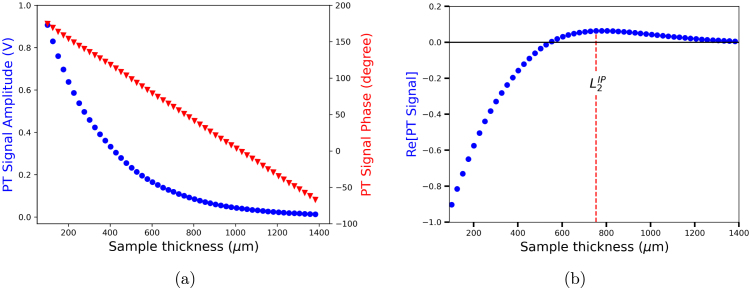
(a) Experimental PT signal amplitude and phase of a deionized water sample, measured at 25 °C, (b) real part of the PT signal.

Assuming that the thermal diffusivity of the sample is unknown, the first measurement involves determining the range of the thickness scan that satisfied the thermally thick condition, explained in Section 6.1. Fig. 11(a) displays the amplitude (A) and phase (ϕ) of the PT signal in a range of the sample thickness (L) from L=100μm to L=1400μm, relative to the position of the piezoelectric sensor. From these data, the real part (Re[Aexp(iϕ)]) is shown in Fig. 11(b). The extreme value observed at the position $L=L_{2}^{IP}\approx 753\;\mu \text{m}$ corresponds to the second critical point, n=2, and using $L_{2}^{IP}=\left(n-\frac{1}{2}\right)\frac{\pi }{2}\mu$, the value of μ is 320μm. Hence, the thermally thick condition starts at 2μ≈640μm.

**Fig. 12:**
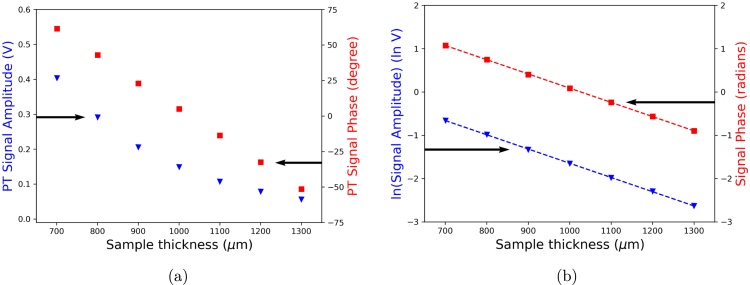
(a) Typical TWRC experimental data for the thermally thick condition, (b) Linearization of the PT signal amplitude and PT signal phase in radians.

Fig. 12 shows a typical PT signal amplitude and phase for a deionized water sample, measured at 25°C, for a thickness scan from 2μ≈700μm to 4μ≈1300μm relative to the position of the piezoelectric sensor, which corresponds to the thermally thick condition. Fig. 12(b) shows the linearization of the PT signal amplitude by computing its natural logarithm and the PT signal phase expressed in radians. Performing a straight line fit to the experimental data (dash lines in Fig. 12(b)), The slopes are used to determine the thermal diffusivity by using the Eq. (4). The thermal diffusivity values were $\alpha_{A}=1.454\times 10^{-7}\;{\text{m s}}^{-1}$ and $\alpha_{\phi }=1.457\times 10^{-7}\;{\text{m s}}^{-1}$, which agree with the value reported in the literature for water at 25°C [25].

Two kinds of experiments were conducted to demonstrate the effectiveness of temperature control. The first approach involved taking measurements at ambient temperature on different days and hours of the day without applying temperature control. Then, a new set of measurements was done with the implemented temperature control at the same time interval. The second procedure consisted of determining the thermal diffusivity of a sample in a controlled temperature interval.

**Fig. 13:**
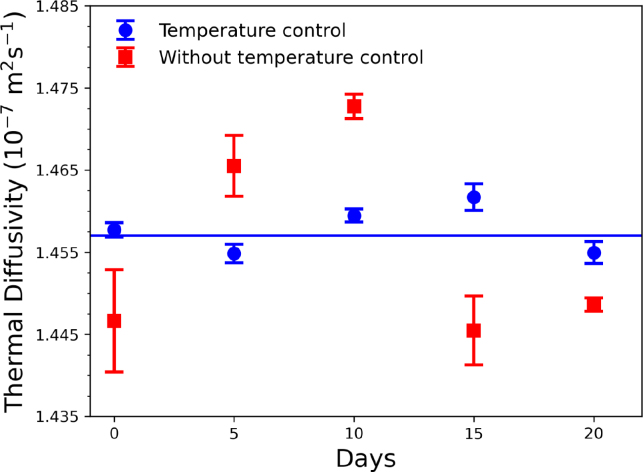
Thermal diffusivity of water measured at different days.

**Fig. 14:**
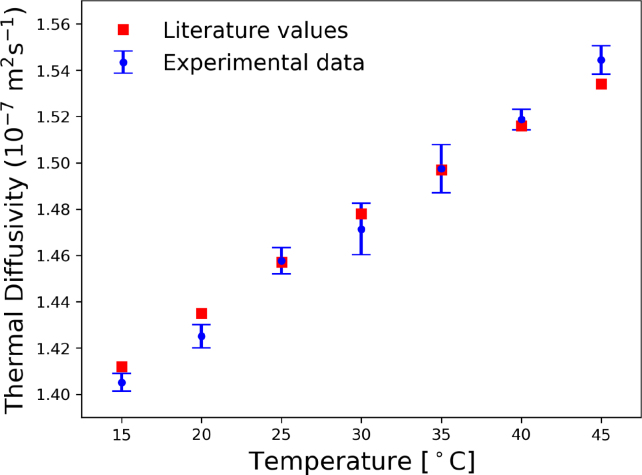
Thermal diffusivity of water measured at different temperatures.

Fig. 13 illustrates the behavior of the thermal diffusivity of deionized water with and without temperature control, measured at different days and hours of the day for twenty days. The thermal diffusivities measured without temperature control show a fluctuation between $1.440\times 10^{-7}\;{\text{m s}}^{-1}$ to $1.475\times 10^{-7}\;{\text{m s}}^{-1}$. This behavior was because the laboratory’s ambient temperature variation during the measurement hours was 25.0±2.5°C. On the other hand, the thermal diffusivity measurements done with the implemented temperature control fix at 25.0 ± 0.1 °C had less fluctuation, (1.457 ± 0.002) × $10^{-7}\;{\text{m s}}^{-1}$. This experiment also demonstrates the reproducibility of the measurements using the TWRC with temperature control and the sensitivity of the TWRC instrument to variations of the sample temperature because the values of the thermal diffusivity in the range of the fluctuated room temperature are consistent with the values reported in the literature [25].

In the second experiment, the thermal diffusivity of deionized water was measured by changing the sample temperature from 15 °C to 45 °C using the implemented temperature control. The results are displayed in Fig. 14 and compared with the values reported in the literature. A good agreement between the experimental data and literature values was obtained [25].

As demonstrated, the implemented temperature control, coupled with the thermal wave resonant cavity setup, helps measure the thermal diffusivity of liquid samples at different temperatures, which is crucial as this thermal property is temperature-dependent. The implementation of this experimental procedure can be helpful in the study of several temperature-dependent phenomena in complex liquid samples, facilitating a more comprehensive understanding of the thermal transport processes occurring within these materials. Additionally, it is recommended to run preliminary measurements using a material with well-known thermal diffusivity, such as deionized water to verify the system’s proper operation and the correct alignment between the laser beam, thermal wave generator, and piezoelectric sensor, before to start a study of a new set of samples.

## CRediT authorship contribution statement

**Miguel Ceja-Morales:** Writing – original draft, Validation, Software, Methodology, Investigation, Conceptualization. **Pedro E. García-González:** Software. **Luis M. Montes-De-Oca:** Writing – review & editing, Methodology, Investigation, Conceptualization. **R.A. Medina-Esquivel:** Writing – review & editing, Validation, Supervision, Methodology, Conceptualization. **Miguel Zambrano-Arjona:** Writing – review & editing, Validation, Methodology. **Nikte M. Gomez-Ortiz:** Writing – review & editing, Methodology. **P. Martínez-Torres:** Writing – review & editing, Supervision, Investigation, Conceptualization.

## Ethics statements

This research project concentrated on the advancement of software and the execution of experimental procedures, eschewing the involvement of human participants, animals, or the utilization of sensitive data. Consequently, the study did not necessitate formal ethical approval.

## Declaration of competing interest

The authors declare that they have no known competing financial interests or personal relationships that could have appeared to influence the work reported in this paper.
